# Cation Induced Structural Variation in Topochemically Modified Dion-Jacobson Perovskite Solid Solutions

**DOI:** 10.3390/molecules30224430

**Published:** 2025-11-16

**Authors:** Roshni Bhuvan, Gary J. Sandrock, Sara Akbarian-Tefaghi, Mya Kelch, Mark Granier, John B. Wiley

**Affiliations:** Department of Chemistry and the Advanced Materials Research Institute, The University of New Orleans, New Orleans, LA 70148, USA; rbhuvan@my.uno.edu (R.B.); gjsandro@my.uno.edu (G.J.S.); sakbari1@uno.edu (S.A.-T.); ckkelch@uno.edu (M.K.); mgranier@uno.edu (M.G.)

**Keywords:** Dion-Jacobson, layered perovskites, ion exchange, solid solutions

## Abstract

The continuous solid solution series based on the ion exchangeable Dion-Jacobson layered perovskites, A_1−x_A′_x_LaNb_2_O_7_ (A/A′ = Li, Na, K, Rb, Cs; 0 ≤ x ≤ 1), has been investigated to illuminate the relationship between composition and structure. Topochemical synthesis of the solid solutions from combinations of various alkali metal cations has been achieved by reacting pure end members (ALaNb_2_O_7_) at appropriate ratios and temperatures. All adjacent sets of alkali metals (Li/Na, Na/K, K/Rb, and Rb/Cs) readily formed solid solutions, while only the one non-adjacent solid solution, K_1−x_Cs_x_LaNb_2_O_7_ (K/Cs), could be obtained. Local cation coordination and the corresponding layer alignments vary as a function of composition where the relative concentration of the larger cation dictates structure. Thermal analysis of the solid solutions, A_1−x_A′_x_LaNb_2_O_7_ (A/A′ = Li, Na, K) showed that the lithium- and sodium-containing compositions were thermally unstable. This study demonstrates that the systematic variation in average cation sizes in the solid solution series allows for structural control in these important perovskite hosts.

## 1. Introduction

**Figure 1:**
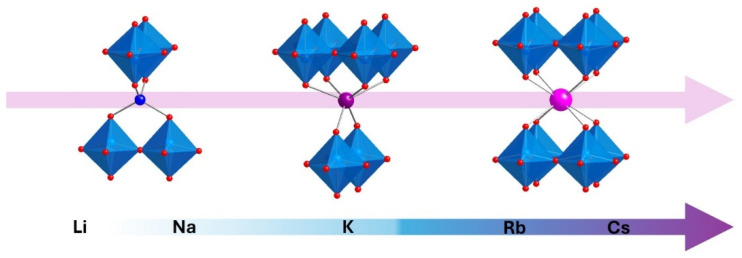
Representations of the various orientations of the perovskite slabs and interlayer cation coordination for the staggered (**left**), partially staggered (**center**), and eclipsed (**right**) configurations.

Dion-Jacobson (DJ) layered perovskites have been extensively studied due to their receptiveness to topochemical manipulation [1,2,3,4,5,6,7,8,9,10]. Especially noteworthy among these compounds are the ion exchangeable double-layered series, ALaNb_2_O_7_ (A = Li, Na, K, Rb, Cs) [11,12,13,14,15,16]. KLaNb_2_O_7_, RbLaNb_2_O_7_, and CsLaNb_2_O_7_ are thermodynamically stable phases accessible at high temperatures (>850 °C) [6,10] while metastable LiLaNb_2_O_7_ and NaLaNb_2_O_7_, known to decompose above 700 °C, can be topotactically synthesized via ion exchange at low temperatures (<400 °C) from alkali metal nitrates [13,17]. Interestingly, the interlayer structure of this series can readily vary depending on the identity of the A cation. There is a clear evolution from the smallest (Li) to the largest (Cs) (Figure 1). The small cations, lithium and sodium, half occupy 4-coordinate tetrahedral positions within the interlayer gallery [13,17], the large cations, rubidium and cesium, fully occupy cubic 8-coordinate positions [11,12], and potassium is unique, half occupying a trigonal prismatic 6-coordinate position [15]. As the interlayer cations vary, the structure can compensate by changing the relative orientation of the perovskite layers and the three distinct orientations are denoted as staggered (S), eclipsed (E), and partially staggered (PS), respectively.

This series of DJ double-layered perovskites are all amenable to topochemical modification. Studies exploring ion exchange, intercalation, and grafting, as well as the more intricate construction of interlayer arrays, are known. Early examples extend beyond the simple alkali metal exchange of these hosts where researchers have prepared compounds with other monovalent, ALaNb_2_O_7_ (A = H, NH_4_, Ag) [1,6], and divalent cations, M_0.5_LaNb_2_O_7_ (M = Fe, Ni, Cu, Ca) [8,18]. Intercalation reactions highlight reductive processes with alkali metal insertion leading to mixed valence compounds (Nb^4+/5+^), A_2_LaNb_2_O_7_ (A = Rb) [11]. Grafting reactions have also been fruitful—inorganic–organic hybrids, RLaNb_2_O_7_ (R = organic), including n-alkoxyl, organophosphoric acids, polyethers, and hydroxyaromatic carboxylic acids have all been reported [19,20,21,22]. Further advances, sometimes involving multistep topochemistry, have resulted in the assembly of extended networks within these hosts such as those seen in (MCl)LaNb_2_O_7_ (M = V, Cr, Mn, Fe, Co, Cu) [5,23], (A_2_Cl)LaNb_2_O_7_ (A = Li, Rb, Cs) [24,25], and (A_2_YH)LaNb_2_O_7_ (A = Rb, Cs; Y = O, S, Se) [26,27].

Solid solutions of a variety of compounds have been well studied. Researchers have used combinations of end members to fine tune properties in a number of technologically significant materials including those with applications in structural engineering [28], electronics [29,30,31], magnetics [32], optics [33,34], and medicine [35,36]. Various perovskites are known to form solid solutions leading to important compounds offering technologically significant variation in electronic and magnetic structures, catalytic activity, and ionic conductivity [21,37,38,39]. Herein, we report on the series of layered perovskite solid solutions, A_1−x_A′_x_LaNb_2_O_7_ (A/A′ = Li, Na, K, Rb, Cs; 0 ≤ x ≤ 1). Combinations of adjacent and non-adjacent end members were explored to illuminate synthetic details, structural variations, and thermal stability. Solid solutions in these DJ layered perovskite series are of interest in that they give insight into compositionally dictated structural variations and the corresponding influence of cation size. Further, understanding cation solubility for different cation combinations will better illuminate structure limitations and metastabilities as influenced by the relative orientations of the adjacent layers. Greater understanding of these parameters is not only important to topochemical manipulation of these hosts but will also allow for a greater tunability of properties including those that influence dielectric and catalytic response.

## 2. Results

**Figure 2:**
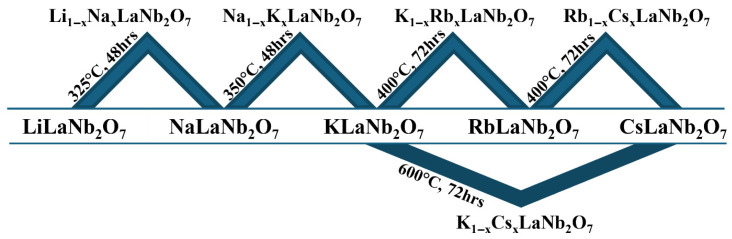
End member combinations that resulted in solid solutions and their corresponding reaction conditions. Other combinations with LiLaNb_2_O_7_ and NaLaNb_2_O_7_ were not successful.

Solid solutions prepared from adjacent cationic species, Li/Na, Na/K, K/Rb, and Rb/Cs, in A_1−x_A′_x_LaNb_2_O_7_ (A/A′ = Li, Na, K, Rb, Cs; 0 ≤ x ≤ 1) and one non-adjacent solid solution, K_1−x_Cs_x_LaNb_2_O_7_ (0 ≤ x ≤ 1), were successfully synthesized topochemically from combinations of the parent compounds ALaNb_2_O_7_ (A = Li, Na, K, Rb, Cs). Ion exchange reactions between adjacent sets of parent compounds readily occurred under mild conditions, ≤400 °C, while the one successful non-adjacent solid solution, K_1−x_Cs_x_LaNb_2_O_7_, required higher temperatures (600 °C). Reactions involving LiLaNb_2_O_7_ or NaLaNb_2_O_7_ with ALaNb_2_O_7_ compounds containing A cations non-adjacent to Li and Na were not effective in that these metastable compounds decomposed before the target solid solutions had a chance to form. Figure 2 and Table S1 summarize the ALaNb_2_O_7_ combinations resulting in the various solid solutions with the corresponding reaction conditions.

**Figure 3:**
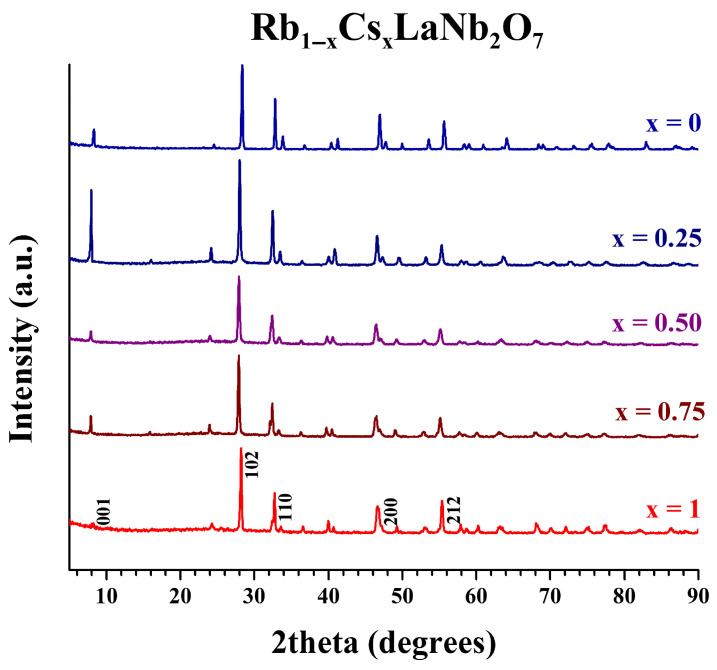
XRD patterns for Rb_1−x_Cs_x_LaNb_2_O_7_ (0 ≤ x ≤ 1) solid solution.

X-ray powder diffraction (XRD) patterns for the Rb_1−x_Cs_x_LaNb_2_O_7_ (0 ≤ x ≤ 1) solid solution are presented in Figure 3 and other members of the series, A_1−x_A′_x_LaNb_2_O_7_ (A/A′ = Li, Na, K, Rb; 0 ≤ x ≤ 1), are shown in Figures S1–S6. Unit cell parameters were refined for the parents (Table S2) and all the solid solutions; Table 1 presents the solid solution series made with adjacent alkali metal cations and Table 2, the solid solution, K_1−x_Cs_x_LaNb_2_O_7_ made with the non-adjacent cations, K and Cs. The XRD patterns for the final solid solutions show highly crystalline, phase-pure products with no evidence of unreacted starting materials or decomposition products. This, along with the narrow low angle diffraction peak that correlates to the interlayer spacing, indicates the successful formation of solid solutions. The influence of the interlayer cation is observed in the longest unit cell parameter; as the interlayer cation increases in size, this parameter increases. (For the compounds A = Rb, Cs, the layer spacing corresponds to the *c*-parameter, for A = Li, Na, this corresponds to half the *c*-parameter, and for the orthorhombic KLaNb_2_O_7_ structure, it is half the *b*-parameter.) The trends for layer expansion across the series of solid solutions are consistent with Vegard’s law [40]. The shorter unit cell parameters (~3.9 Å) for all compounds synthesized stay essentially constant.

**Figure 4:**
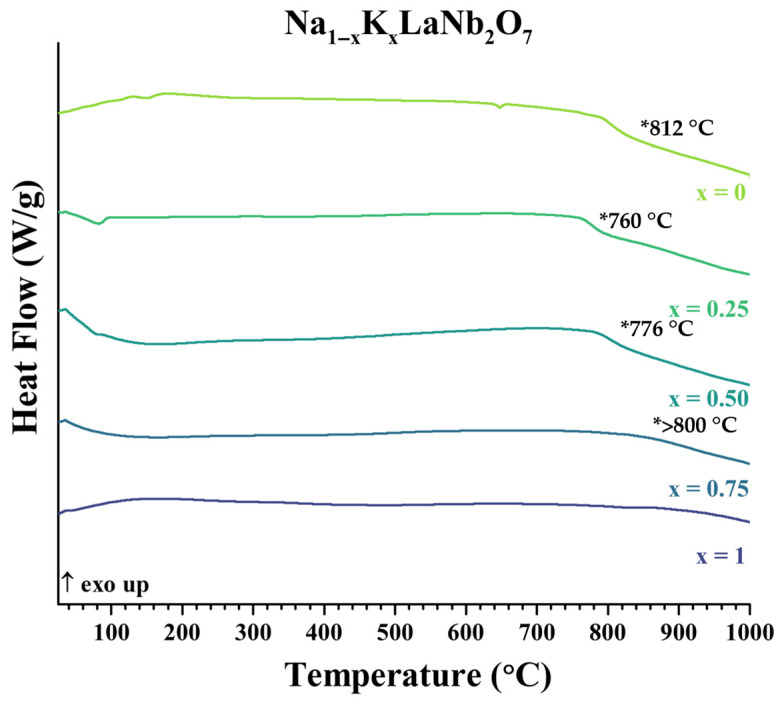
DSC data of the Na_1−x_K_x_LaNb_2_O_7_ (0 ≤ x ≤ 1) series from room temperature to 1000 °C at a heating rate of 5 °C/min under Ar atmosphere. Decomposition temperatures (*) are shown for x = 0. 0.25, 0.50, and 0.75. Small low temperature endotherms (<200 °C, x = 0, 0.25) are due to the loss of water of hydration [17] and the endotherm at 680 °C (x = 0) has been attributed to a structure change in the perovskite layer [17].

The parent compounds, ALaNb_2_O_7_ (A = Li, Na, K) (Figure S7), and Li_1−x_Na_x_LaNb_2_O_7_ (Figure S8) and Na_1−x_K_x_LaNb_2_O_7_ (Figure 4) solid solution series were examined by thermal analysis. The solid solutions showed some metastability through the range of temperatures evaluated and their final decomposition temperatures are tabulated (Table 3). Decomposition products were determined after the differential scanning calorimetry (DSC) measurements via XRD (Figures S9 and S10a). For the more potassium-rich samples, Na_0.5_K_0.5_LaNb_2_O_7_ and Na_0.25_K_0.75_LaNb_2_O_7_, decomposition products were less pronounced. Careful analysis before and after heating the samples to 1000 °C with HTXRD showed the emergence of KLaNb_2_O_7_; this was especially illuminated by the shift in the 0 10 0 reflection (Figure S10b,c). Thermal data show that increasing amounts of Li in Li_1−x_Na_x_LaNb_2_O_7_ and increasing amounts of Na in Na_1−x_K_x_LaNb_2_O_7_ decreases decomposition temperatures.

The compounds, RbLaNb_2_O_7_ and CsLaNb_2_O_7_, are synthesized above 1000 °C and do not show instability at this temperature. The stability of K_1−x_Rb_x_LaNb_2_O_7_, K_1−x_Cs_x_LaNb_2_O_7_, and Rb_1−x_Cs_x_LaNb_2_O_7_ (x = 0.5) were examined up to 1000 °C in air. No evidence for thermal instability, including immiscibility, was observed.

**Figure 5:**
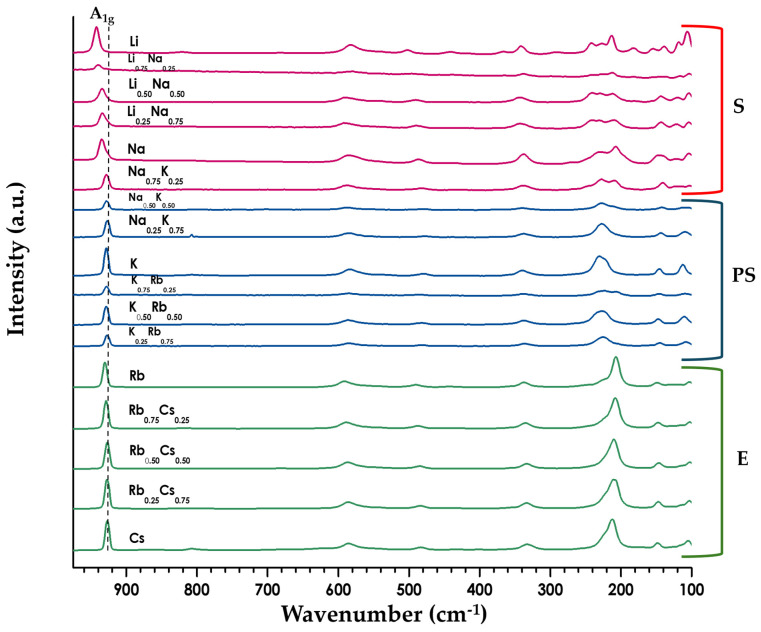
Stack plot of Raman data for the series A_1−x_A′_x_LaNb_2_O_7_ (A/A′ = Li, Na, K, Rb, Cs; 0 ≤ x ≤ 1). Spectra colors vary with layer orientation: green (E), blue (PS), and red (S). Dashed line highlights the blue shift in the A_1g_ vibration for the smaller alkali cations (see Table S3).

Raman spectroscopy was carried out on the parent compounds (Figure S11) and the series of A_1−x_A′_x_LaNb_2_O_7_ solid solutions (Figure 5 and Figures S12–S16). Clear differences can be seen for the parents below 700 cm^−1^ between the S, PS, and E structures (Figure 5). The solid solutions themselves exhibit a fairly consistent spectra within a particular structure type. The higher energy vibrational mode around 930 cm^−1^ did show a shift in energy as a function of cation (dashed line in Figure 5 and Table S3). This peak has been associated with the apical Nb-O bond (A_1g_) of the NbO_6_ octahedra [2,22,41] and it is this oxygen that bonds directly to the alkali metal cations; the vibrational energy is relatively constant for the larger cations, but increases for both Li and Na.

## 3. Discussion

A series of solid solutions were made from the DJ double-layered perovskites, ALaNb_2_O_7_ (A = Li, Na, K, Rb, Cs). The adjacent cation sets of solid solutions were readily prepared: Li/Na (325 °C, 48 h), Na/K (350 °C, 48 h), K/Rb (400 °C, 72 h), and Rb/Cs (400 °C, 72 h). Cation mobilities for ALaNb_2_O_7_ (A = Li, Na, K) have been found to be 10^−3^–10^−4^ S-K/cm around 350 °C [42]. Though we are unaware of any reports on ALaNb_2_O_7_ (A = Rb, Cs) cation mobility, Sato and coworkers reported on the quadruple layer (4L) perovskite ACa_2_NaNb_4_O_13_ (A = Na, Rb) and found that NaCa_2_NaNb_4_O_13_ has similar conductivity to NaLaNb_2_O_7_ at 400 °C while the RbCa_2_NaNb_4_O_13_ is about two orders of magnitude less conductive [43]. It is expected that similar lower cation mobilities are present in ALaNb_2_O_7_ (A = Rb, Cs) and this likely contributes to the need for slightly higher temperatures and longer reaction times for the formation of solid solutions with these cations.

Synthesis of solid solutions from non-adjacent cation sets Li/K, Li/Rb, Li/Cs, Na/Rb, Na/Cs, and K/Cs was more challenging. While the set K_1−x_Cs_x_LaNb_2_O_7_ was accessible, it required a higher temperature (600 °C) to achieve single-phase products. It is expected that the opposing layer orientations (PS vs. E) and the likely lower cationic mobility for Cs, contributed to this synthetic behavior. The solid solutions of the other sets, Li/K, Li/Rb, Li/Cs, Na/Rb, and Na/Cs, could not be prepared. Efforts here also investigated reaction conditions up to 600 °C. This crossed into the metastability region of the Li and Na compounds [10,13,17], resulting in decomposition of these reactants before any solid solutions could form. It is likely that the instability was enhanced due to cation size mismatch for Li and Na with the larger cations, K, Rb, and Cs.

**Figure 6:**
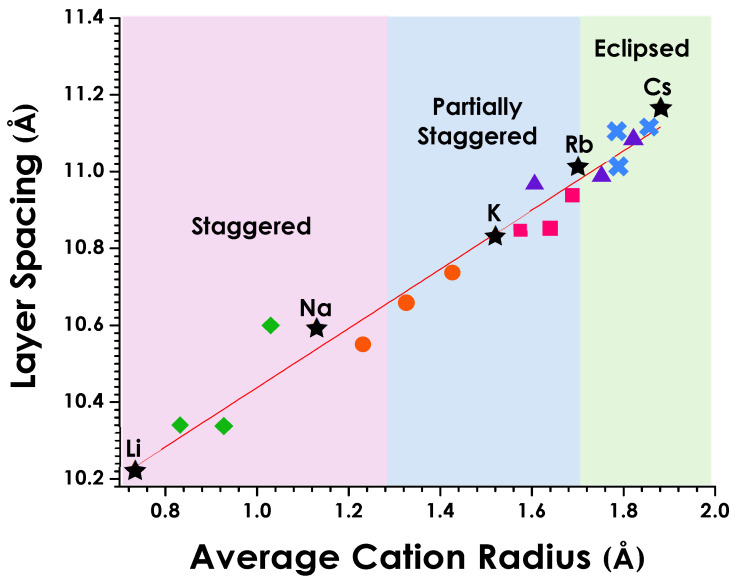
Variation in layer spacing as a function of average cation radius (trend line shown in red; R^2^ = 0.97, calculated by a weighted least-squares method). Shaded regions highlight the staggered (S), partially staggered (PS), and eclipsed (E) orientations of the layers. The parent compounds are indicated by stars (★), Li_1−x_Na_x_LaNb_2_O_7_ solid solution (x = 0.25, 0.50, 0.75) by green diamonds (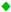), Na_1−x_K_x_LaNb_2_O_7_ by orange circles (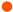), K_1−x_Rb_x_LaNb_2_O_7_ by pink squares (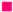), K_1−x_Cs_x_LaNb_2_O_7_ by purple triangles (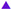), and Rb_1−x_Cs_x_LaNb_2_O_7_ by blue crosses (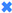).

A clear correlation between layer spacing and cationic radius can be seen for the series of solid solutions (Figure 6). Unit cell parameters were scaled to Z = 1 for the series to allow a direct comparison of corresponding structural elements (i.e., layer spacings). Shannon radii were used to determine the ionic radii for each four, six, and eight coordinate ions [44]. The various ionic radii for the solid solutions were calculated based on a weighted average of the ionic radii (r_ionic_) using the equation:

r_ionic_ = (1 − x)r_A_ + (x)r_A′_ (1)

where r_A_ and r_A′_ are the Shannon radii for the various cations in their respective coordination environments. The cation sizes corresponding to regions that are staggered, partially staggered, and eclipsed layers are color-coded.

As would be expected, the layer spacing for the series of solid solutions increases as larger cations replace smaller ones (Table 1 and Table 2). Figure 6 highlights this behavior showing a continuous variation in layer spacing as a function of cation size. The trend is linear. Early efforts in this study did see some deviation due to the easily hydrated sodium containing compounds, but this impact was mediated by obtaining data on slightly heated samples. As can be seen by the series of solid solutions, transitions from one layer orientation to another, staggered (S) to partially staggered (PS) to eclipsed (E), are clearly influenced by cation sizes. The transitions occur at average sizes of approximately 1.3 Å (S → PS) and 1.7 Å (PS → E) for the sets of alkali metal cation solid solutions.

There are many other known A substituents in ALaNb_2_O_7_ such that one can consider the influence of cation size and layer spacing on the observed structures for these various exchange products. The silver compounds, β-AgLaNb_2_O_7_ and α-AgLaNb_2_O_7_, for example, favor the staggered conformation as expected based on the silver cation size of 1.14 Å [44] and layer spacings of 10.647 Å and 10.679 Å, respectively [1]. For the ammonium compound, NH_4_LaNb_2_O_7_, the layer spacing of 10.95 Å is consistent with its eclipsed conformation [6]; however, from the NH_4_^+^ cation sizes reported, 1.48 Å (CN 6) and 1.54 Å (CN 8) [44], one would expect the PS layer orientation. The tetrahedral shape of the polyatomic cation and hydrogen bonding may be important here in leading to the eclipsed conformation. In anhydrous HLaNb_2_O_7_, the interaction of the cation is more localized and, despite the extremely small size of the H^+^, an eclipsed layer orientation is observed with a very small layer spacing c ≈ 10.46 Å. This structure is favored due to the hydrogen covalently linked to an apical oxygen in one perovskite layer while bridging (hydrogen bonding) to an apical oxygen of the adjacent perovskite layer (OꟷHꞏꞏꞏꞏO) [45,46].

Divalent cation exchange products (M_0.5_LaNb_2_O_7_) are also known [8,18] and these do not follow the same trends seen for the alkali metal solid solutions. While staggered configurations would be predicted based on both cation sizes and interlayer spacings, the transition metal compounds (M = Fe, Ni, Cu) have eclipsed structures [8] while the calcium compound (M = Ca) is partially staggered [18]. Detailed structure refinements have not been reported for these compounds, but it is expected that in the case of the transition metals, the eclipsed configuration is driven by covalent interaction of these metals with perovskite layer oxygens.

Thermal analysis of the solid solution series focused on those compounds, Li_1− x_Na_x_LaNb_2_O_7_ (Figure S8) and Na_1−x_K_x_LaNb_2_O_7_ (Figure 4), derived from the metastable parents, LiLaNb_2_O_7_ and NaLaNb_2_O_7_. Thermal instability varied for the Li_1−x_Na_x_LaNb_2_O_7_ series where an increase in lithium content was associated with a lower decomposition temperature, decreasing from 812 °C for NaLaNb_2_O_7_ to 719 °C for LiLaNb_2_O_7_ [10,13,17]. Interestingly, the drop in decomposition temperature was most dramatic with the introduction of only 25% Li (Table 3). In the Li_1−x_Na_x_LaNb_2_O_7_ series, decomposition products were found to be LaNbO_4_ and ANbO_3_ (A = Li, Na) (Figure S9). One might expect corresponding solid solutions, Li_1−x_Na_x_NbO_3_, but the LiNbO_3_-NaNbO_3_ phase diagram exhibits an immiscibility dome favoring the separate phases with both low and high x values [47]. The Na_1−x_K_x_LaNb_2_O_7_ was also found to be unstable. The decomposition of Na_0.75_K_0.25_LaNb_2_O_7_ (x = 0.25) leads to LaNbO_4_ and NaNbO_3_ as well as the starting material, KLaNb_2_O_7_ (Figure S10a). Decompositions for those compounds with a higher potassium content (x = 0.50 and 0.75) were found to be more subtle in terms of the byproducts (Figure S10b,c); loss of the solid solution was best revealed by the expansion of the layer spacing corresponding to the appearance of KLaNb_2_O_7_.

Raman spectroscopy was used to examine the local structure of the series of layered perovskites (Figure 5 and Figures S11–S16, Table S3). The band around 930 cm^−1^, which corresponds to the stretch of the short Nb-O outer apical oxygen, helps to illuminate the interlayer structure; changes in the vibrational energy are associated with a change in bond order for the Nb-O stretch. For the Ruddlesden-Popper series, A_2_La_2_Ti_3_O_10_ (A = Na, K, or Rb), it is found that as the cation size increases there is a corresponding red shift indicating a decrease in bond order [2]. In contrast, the Dion-Jacobson series, ALaNb_2_O_7_ (A = Na, K, Rb, Cs), shows minimal variation in vibrational energy, remaining at about 930 cm^−1^ for this series (Figure 5 and Table S3). We see similar behavior for the solid solutions, A_1−x_A′_x_LaNb_2_O_7_ (A/A′ = Na, K, Rb; 0 ≤ x ≤ 1). The one exception is seen with the lithium compounds where the A_1g_ band blue shifts up to 943 cm^−1^, indicating a much higher Nb-O bond order for the Li_1−x_Na_x_LaNb_2_O_7_ series likely due to the smaller nonpolarizable lithium cations.

Adjacent cation sets and one non-adjacent set readily form solid solutions with the interlayer spacing and layer orientations varying continuously with cation size. No evidence for cation site segregation was observed even when there is an evolution in layer orientations, S → PS → E. Combinations of exchange ions beyond or including alkali metals may be needed to achieve this where deep local minima are needed to induce extensive cation segregation favoring alternating layered superstructures. Interest in these compounds, and their possible segregated layers, is due to their assumed reactivity differences so that topochemical manipulation can be layer-specific, possibly leading to new intricate layered architectures exhibiting cooperative electronic and/or magnetic properties.

## 4. Materials and Methods

**Synthesis.** Parent Compounds: Synthesis of the parent compounds in this study was performed following methods similar to Sato and colleagues [13,15,42]. Parent compounds, ALaNb_2_O_7_ (A = Rb, Cs), were prepared with appropriate molar quantities of starting reagents La_2_O_3_ (Alfa Aesar (Ward Hill, MA, USA), 99.99%), Nb_2_O_5_ (Alfa Aesar, 99.9985%), and 20% molar excess of Rb_2_CO_3_ (Alfa Aesar, 99.975%) or Cs_2_CO_3_ (Alfa Aesar, 99.994%). La_2_O_3_ and Nb_2_O_5_ were pretreated at 1000 °C for 12–24 h prior to reaction to remove any carbonates, hydroxides, or nonstoichiometry. The measured reagents were ground for 30 min in an agate mortar and pestle and transferred to alumina crucibles (Coors (Golden, CO, USA) AD-998). RbLaNb_2_O_7_ was reacted at 850 °C for 24 h, then 1050 °C for 24 h with grinding between annealing; CsLaNb_2_O_7_ was reacted at 1050 °C for 48 h with intermittent grinding. Both samples were washed post-reaction with distilled water and acetone, with centrifugation between washes, to remove any unreacted carbonates. Samples were allowed to dry fully overnight at 120 °C, resulting in crystalline white powders.

The parent compounds ALaNb_2_O_7_ (A = Li, Na, K) were synthesized by ion exchange from RbLaNb_2_O_7_. A stoichiometric amount of RbLaNb_2_O_7_ was ground together with a ten-times molar excess of LiNO_3_ (Alfa Aesar, 99%), NaNO_3_ (Alfa Aesar 99.999%), or KNO_3_ (Alfa Aesar, 99.994%) for 30 min in an agate mortar and pestle. The samples were reacted in alumina boats at 300 °C, 360 °C, and 380 °C for 72 h, for the synthesis of LiLaNb_2_O_7_, NaLaNb_2_O_7_, and KLaNb_2_O_7_, respectively. The products were then washed with distilled water and acetone, centrifuging between washes. The resulting white powders were allowed to dry fully in a 120 °C drying oven. Note: NaLaNb_2_O_7_ forms a hydrate when exposed to air. To minimize water uptake, samples were left in the drying oven at 120 °C until needed for reactions or testing.

Reaction conditions for the series ALaNb_2_O_7_ (A = Li, Na, K, Rb, Cs) are summarized in Table S1. Figures S1 and S2 show the various X-ray powder patterns of this series compared to each other and to reference patterns. Table S2 presents refined unit cell parameters and cell volumes. All the parent compounds are in good agreement with the literature, and the powder patterns show the samples to be highly crystalline, pure products.

Solid Solutions. Synthesis of the solid solutions, A_1−x_A′_x_LaNb_2_O_7_ (A/A′ = Li, Na, K, Rb, Cs), was accomplished through stoichiometric combinations of various parent compounds. Adjacent cation pairs (Li/Na, Na/K, K/Rb, and Rb/Cs) were all prepared with compositions of x = 0.25, 0.50, and 0.75. These were ground together for 30 min in air in an agate mortar and pestle, pelleted (7 mm diameter with a Sigma-Aldrich (St. Louis, MO, USA) quick press), and reacted in an alumina crucible at 325–400 °C for 48–72 h (temperatures and duration varied with the solid solution); Li_1−x_Na_x_LaNb_2_O_7_ (x = 0.25, 0.50, 0.75) was synthesized at 325 °C and Na_1−x_K_x_LaNb_2_O_7_ (x = 0.25, 0.50, 0.75) at 350 °C, both for 48 h. K_1−x_Rb_x_LaNb_2_O_7_ (x = 0.25, 0.50, 0.75) and Rb_1−x_Cs_x_LaNb_2_O_7_ (x = 0.25, 0.50, 0.75) were prepared at 400 °C for 72 h. Reaction conditions for the series A_1−x_A′_x_LaNb_2_O_7_ (A/A′ = Li, Na, K, Rb, Cs) are summarized in Table S1.

Solubility in non-adjacent pairs was initially examined at the x = 0.50 composition under various reaction conditions. The K/Cs combination reacted but it was found that temperatures > 500 °C were needed to obtain a single-phase product. The combinations of Li/K, Li/Rb, Li/Cs, Na/Rb, and Na/Cs were explored at various reaction temperatures up to 600 °C without the formation of solid solutions; this instead resulted in either no reaction at lower temperatures or decomposition of LiLaNb_2_O_7_ and NaLaNb_2_O_7_ to LiNbO_3_ with LaNbO_4_ and NaNbO_3_ with LaNbO_4_, respectively. The one successful non-adjacent solid solution, K_1−x_Cs_x_LaNb_2_O_7_ (x = 0.25, 0.50, 0.75), was obtained at 600 °C in 72 h.

**Characterization.** X-ray powder diffraction (XRD) data were collected on a Panalytical system equipped with Cu-Kα radiation (λ = 1.5418 Å) and a curved graphite monochromator. Scans were conducted in continuous mode with a rate of 0.5 °/s. The peak positions and lattice parameters were refined using a least-squares method with the CrystalDiffract 7.0 program [48]. Sodium containing compounds were found to readily absorb water; to obtain anhydrous diffraction data on sodium-rich solid solutions, samples were heated to 300 °C on an Anton Paar HTK1600 high temperature stage (Ashland, VA, USA). Thermogravimetric analysis (TGA) and differential scanning calorimetry (DSC) data were obtained using a TA Instruments TGA-DSC SDT Q600 system (New Castle, DE, USA) in alumina pans under pure argon, where samples were heated up to 1000 °C with a rate of 5 °C/min. Raman spectra of samples in capillary tubes were collected on a Thermo-Fisher DXR dispersive Raman spectrometer (Waltham, MA, USA) with a wavelength of 532 nm and a spectral resolution of 3 cm^−1^.

## 5. Conclusions

A continuous solid solution series based on the Dion-Jacobson (DJ) ion exchangeable layered perovskites, A_1−x_A′_x_LaNb_2_O_7_ (A/A′ = Li, Na, K, Rb, Cs; 0 ≤ x ≤ 1), has been investigated to determine the relationship of the solid solution composition and the structure of the final products. Solid solution synthesis has been achieved by topochemically reacting pure end members under the appropriate ratios and temperatures. All adjacent alkali metals (Li/Na, Na/K, K/Rb, Rb/Cs) were accessible, while only the non-adjacent K/Cs solid solution could be obtained. Average interlayer cation sizes direct the final structure of the material, and in solid solutions the larger cation dictates the structure with increasing x values. Insight into these solid solutions allows researchers to expand the library of precursors available for topochemical manipulation, possibly leading to new technologically significant materials including those pertinent to cooperative properties as well as dielectric and catalytic activities.

## Figures and Tables

**Table 1:** Refined unit cell parameters for perovskite solid solutions, A_1−x_A′_x_LaNb_2_O_7_ (A/A′ = Li, Na, K, Rb, Cs; 0 ≤ x ≤ 1), prepared with adjacent alkali cations.

Unit Cell Parameters for A_1−x_A′_x_LaNb_2_O_7_ (A/A′ = Li, Na, K, Rb, Cs; 0 ≤ x ≤ 1)								
A	x	a (Å)	b (Å)	c (Å)	Unit Cell *	Layer Spacing (Å)	V (Å^3^)	Normalized V (Å^3^) **
**Li**		3.893(2)	3.893(2)	20.435(8)	t	10.218	309.8(2)	154.9
**Li_1−x_Na_x_**	**0.25**	3.919(2)	3.919(2)	20.649(8)	t	10.325	317.2(3)	158.6
	**0.50**	3.92(1)	3.92(1)	20.65(5)	t	10.33	317(2)	159
	0.75 ***	3.90(1)	3.90(1)	21.18(6)	t	10.59	322(1)	161
Na **		3.900(4)	3.900(4)	21.183(2)	t	10.592	322.2(6)	161.1
**Na_1−x_K_x_**	0.25 ***	3.95(6)	3.95(6)	21.1(3)	t	10.55	329(8)	164.6
	**0.50**	3.901(7)	21.312(4)	3.875(7)	o	10.656	322.10(1)	161.05
	**0.75**	3.906(7)	21.455(5)	3.881(7)	o	10.728	325.28(1)	162.64
**K**		3.899(1)	21.658(8)	3.888(1)	o	10.829	328.4(2)	164.2
**K_1−x_Rb_x_**	**0.25**	3.895(2)	21.706(1)	3.888(2)	o	10.853	328.73(2)	164.37
	**0.50**	3.898(4)	21.698(3)	3.887(4)	o	10.849	328.68(7)	164.34
	**0.75**	3.897(1)	21.890(6)	3.882(1)	o	10.945	331.2(2)	165.6
**Rb**		3.886(3)	3.886(3)	10.991(1)	t	10.991	165.95(3)	165.95
**Rb_1−x_Cs_x_**	**0.25**	3.889(5)	3.889(5)	11.016(2)	t	11.016	166.57(4)	166.57
	**0.50**	3.895(7)	3.895(7)	11.086(2)	t	11.086	168.16(6)	168.16
	**0.75**	3.897(6)	3.897(6)	11.119(2)	t	11.119	168.83(5)	168.83
**Cs**		3.906(1)	3.906(1)	11.160(3)	t	11.160	170.26(8)	170.26

* Unit cells: t = tetragonal or o = orthorhombic; ** Cell volumes normalized to Z = 1; *** XRD of sample was taken at 300 °C to avoid hydration.

**Table 2:** Refined unit cell parameters for perovskite solid solution, K_1−x_Cs_x_LaNb_2_O_7_ (0 ≤ x ≤ 1), prepared with the non-adjacent alkali cations.

Unit Cell Parameters for K_1−x_Cs_x_LaNb_2_O_7_ (0 ≤ x ≤ 1)								
A	x	a (Å)	b (Å)	c (Å)	Unit Cell *	Layer Spacing (Å)	V (Å^3^)	Normalized V (Å^3^) **
**K**		3.899(1)	21.658(8)	3.888(1)	o	10.829	328.4(2)	164.2
**K_1−x_Cs_x_**	**0.25**	3.905(2)	21.93(1)	3.892(2)	o	10.97	333.2(3)	166.6
	**0.50**	3.8909(4)	3.8909(4)	11.011(1)	t	11.011	166.69(3)	166.69
	**0.75**	3.8984(3)	3.8984(3)	11.091(1)	t	11.091	168.55(2)	168.55
**Cs**		3.906(1)	3.906(1)	11.160(3)	t	11.160	170.26(8)	170.26

* Unit cells: t = tetragonal or o = orthorhombic; ** Cell volumes normalized to Z = 1.

**Table 3:** Decomposition temperatures and products formed after DSC of Li_1−x_Na_x_LaNb_2_O_7_ and Na_1−x_K_x_LaNb_2_O_7_ (0 ≤ x ≤ 1) series.

Compound		Decomposition Temperature (°C)	Decomposition Products
**LiLaNb_2_O_7_**		719	LiNbO_3_, LaNbO_4_
**Li_1−x_Na_x_**	**0.25**	725	LiNbO_3_, NaNbO_3_, LaNbO_4_
	**0.50**	734	LiNbO_3_, NaNbO_3_, LaNbO_4_
	**0.75**	752	LiNbO_3_, NaNbO_3_, LaNbO_4_
**NaLaNb_2_O_7_**		812	NaNbO_3_, LaNbO_4_
**Na_1−x_K_x_**	**0.25**	760	KLaNb_2_O_7_, NaNbO_3_, LaNbO_4_
	**0.50**	773	KLaNb_2_O_7_, NaNbO_3_, (LaNbO_4_)
	**0.75**	>800	KLaNb_2_O_7_, NaNbO_3_, (LaNbO_4_)
**KLaNb_2_O_7_**		No evidence of decomposition up to 1000 °C	

## Data Availability

The data presented in this study are available in the article and Supplementary Materials.

## Supplementary Materials

The following supporting information can be downloaded at https://www.mdpi.com/article/10.3390/molecules30224430/s1, Figure S1: X-ray diffraction data for ALaNb_2_O_7_ for (a) LiLaNb_2_O_7_, (b) NaLaNb_2_O_7_, (c) KLaNb_2_O_7_, (d) RbLaNb_2_O_7_, and (e) CsLaNb_2_O_7_; Figure S2: X-ray diffraction data for parent compounds, ALaNb_2_O_7_ (A = Li, Na, K, Rb, Cs); Figure S3: X-ray diffraction data for Li_1−x_Na_x_LaNb_2_O_7_ (0 ≤ x ≤ 1) solid solution series; Figure S4: X-ray diffraction data for Na_1−x_K_x_LaNb_2_O_7_ (0 ≤ x ≤ 1) solid solution series; Figure S5: X-ray diffraction data for K_1−x_Rb_x_LaNb_2_O_7_ (0 ≤ x ≤ 1) solid solution series; Figure S6: X-ray diffraction data for K_1−x_Cs_x_LaNb_2_O_7_ (0 ≤ x ≤ 1) solid solution series; Figure S7: Differential scanning calorimetry (DSC) data for parent compounds ALaNb_2_O_7_ (A = Li, Na, K) from room temperature to 1000 °C; Figure S8: DSC data of Li_1−x_Na_x_LaNb_2_O_7_ solid solution series (0 ≤ x ≤ 1) from room temperature to 1000 °C; Figure S9: X-ray powder diffraction data for decomposition products for the series Li_1−x_Na_x_LaNb_2_O_7_ These are shown versus reference patterns for LiNbO_3_, NaNbO_3_ and LaNbO_4_; Figure S10: (a) X-ray powder diffraction data for decomposition products for Na_0.75_K_0.25_LaNb_2_O_7_ relative to NaNbO_3_, LaNbO_4_, and KLaNb_2_O_7_. (b, c) Diffraction data from HTXRD at 500 °C, before and after heating to 1000 °C, for (b) Na_0.50_K_0.50_LaNb_2_O_7_ and (c) Na_0.25_K_0.75_LaNb_2_O_7_.; Figure S11: Raman spectra for parent compounds ALaNb_2_O_7_ (A = Li, Na, K, Rb, Cs); Figure S12: Raman spectra for Li_1−x_Na_x_LaNb_2_O_7_ (0 ≤ x ≤ 1) solid solution series; Figure S13: Raman spectra for Na_1−x_K_x_LaNb_2_O_7_ (0 ≤ x ≤ 1) solid solution series; Figure S14: Raman spectra for K_1−x_Rb_x_LaNb_2_O_7_ (0 ≤ x ≤ 1) solid solution series; Figure S15: Raman spectra for K_1−x_Cs_x_LaNb_2_O_7_ (0 ≤ x ≤ 1) solid solution series; Figure S16: Raman spectra of Rb_1−x_Cs_x_LaNb_2_O_7_ (0 ≤ x ≤ 1) solid solution series; Table S1: Reaction conditions for parent compounds ALaNb_2_O_7_ (A = Li, Na, K, Rb, Cs) and solid solutions A_1−x_A′_x_LaNb_2_O_7_ (A/A′ = Li, Na, K, Rb, Cs); Table S2: Unit cell parameters for ALaNb_2_O_7_ (A = Li, Na, K, Rb, Cs) parent compounds; Table S3: Summary of Raman data of apical Nb-O bond within the A_1−x_A′_x_LaNb_2_O_7_ (A/A′ = Li, Na, K, Rb, Cs; 0 ≤ x ≤ 1) solid solutions compounds.

## Abbreviations

The following abbreviations are used in this manuscript:

DJ	Dion-Jacobson
S	Staggered
E	Eclipsed
PS	Partially staggered
XRD	X-ray powder diffraction
DSC	Differential scanning calorimetry
