# Supplementary material for: Cation Induced Structural Variation in Topochemically Modified Dion-Jacobson Perovskite Solid Solutions
Source: Molecules. 2025 Nov 16;30(22):4430. doi: 10.3390/molecules30224430 (PMC12655364; doi:10.3390/molecules30224430)
Supplement: Supplementary file 1 [file molecules-30-04430-s001.zip › molecules-3919917-supplementary.pdf]

Supplementary Information

Cation Induced Structural Variation in Topochemically Modified Dion-Jacobson Perovskite Solid Solutions

Roshni Bhuvan, Gary J. Sandrock, Sara Akbarian-Tefaghi, Mya Kelch, Mark Granier, and John B. Wiley*

Department of Chemistry and the Advanced Materials Research Institute, The University of New Orleans, New Orleans, LA 70148 USA

Figures

X-ray diffraction data

Figure S1. X-ray diffraction data for $ALaNb_2O_7$ for (a) $LiLaNb_2O_7$, (b) $NaLaNb_2O_7$, (c) $KLaNb_2O_7$, (d) $RbLaNb_2O_7$, and (e) $CsLaNb_2O_7$.

Figure S2. X-ray diffraction data for parent compounds, $ALaNb_2O_7$ (A = Li, Na, K, Rb, Cs).

Figure S3. X-ray diffraction data for $Li_{1-x}Na_xLaNb_2O_7$ ($0 \leq x \leq 1$) solid solution series.

Figure S4. X-ray diffraction data for $Na_{1-x}K_xLaNb_2O_7$ ($0 \leq x \leq 1$) solid solution series.

Figure S5. X-ray diffraction data for $K_{1-x}Rb_xLaNb_2O_7$ ($0 \leq x \leq 1$) solid solution series.

Figure S6. X-ray diffraction data for $K_{1-x}Cs_xLaNb_2O_7$ ($0 \leq x \leq 1$) solid solution series.

Differential scanning calorimetry data

Figure S7. Differential scanning calorimetry (DSC) data for parent compounds $ALaNb_2O_7$ (A = Li, Na, K) from room temperature to 1000 °C.

Figure S8. DSC data of $Li_{1-x}Na_xLaNb_2O_7$ solid solution series ($0 \leq x \leq 1.0$) from room temperature to 1000 °C.

X-ray diffraction data for thermal analysis decomposition products

Figure S9. X-ray powder diffraction data for decomposition products for the series $Li_{1-x}Na_xLaNb_2O_7$. These are shown versus reference patterns for $LiNbO_3$, $NaNbO_3$ and $LaNbO_4$.

Figure S10. (a) X-ray powder diffraction data for decomposition products for $Na_{0.75}K_{0.25}LaNb_2O_7$ relative to $NaNbO_3$, $LaNbO_4$, and $KLaNb_2O_7$. (b, c) Diffraction data from HTXRD at 500 °C, before and after heating to 1000 °C, for (b) $Na_{0.50}K_{0.50}LaNb_2O_7$ and (c) $Na_{0.25}K_{0.75}LaNb_2O_7$.

Raman data

Figure S11. Raman spectra for parent compounds $ALaNb_2O_7$ (A = Li, Na, K, Rb, Cs).

Figure S12. Raman spectra for $Li_{1-x}Na_xLaNb_2O_7$ ($0 \leq x \leq 1$) solid solution series.

Figure S13. Raman spectra for $Na_{1-x}K_xLaNb_2O_7$ ($0 \leq x \leq 1$) solid solution series.

Figure S14. Raman spectra for $K_{1-x}Rb_xLaNb_2O_7$ ($0 \leq x \leq 1$) solid solution series.

Figure S15. Raman spectra for $K_{1-x}Cs_xLaNb_2O_7$ ($0 \leq x \leq 1$) solid solution series.

Figure S16. Raman spectra of $Rb_{1-x}Cs_xLaNb_2O_7$ ($0 \leq x \leq 1$) solid solution series.

Tables

Table S1. Reaction conditions for parent compounds $ALaNb_2O_7$ (A = Li, Na, K, Rb, Cs) and solid solutions $A_{1-x}A'_xLaNb_2O_7$ (A/A' = Li, Na, K, Rb, Cs).

Table S2. Unit cell parameters for $ALaNb_2O_7$ (A = Li, Na, K, Rb, Cs) parent compounds.

Table S3. Summary of Raman data of apical Nb-O bond within the $A_{1-x}A'_xLaNb_2O_7$ (A/A′ = Li, Na, K, Rb, Cs, $0 \leq x \leq 1$) solid solutions compounds.

1. Synthetic Conditions

Table S1. Reaction conditions for parent compounds $ALaNb_2O_7$ (A = Li, Na, K, Rb, Cs) and solid solutions $A_{1-x}A'_xLaNb_2O_7$ (A/A' = Li, Na, K, Rb, Cs).

Compound		Reaction Method	Reaction Conditions (°C, h)*
**$LiLaNb_2O_7$**		Ion Exchange from $RbLaNb_2O_7$ + $LiNO_3$	325°C, 72 h
**$Li_{1-x}Na_x$**	**0.25**	Synthesis using stoichiometric amounts of $LiLaNb_2O_7$ and $NaLaNb_2O_7$	325°C, 72 h
	**0.50**		
	**0.75**		
**$NaLaNb_2O_7$**		Ion Exchange from $RbLaNb_2O_7$ + $NaNO_3$	350°C, 72 h
**$Na_{1-x}K_x$**	**0.25**	Synthesis using stoichiometric amounts of $NaLaNb_2O_7$ and $KLaNb_2O_7$	350°C, 72 h
	**0.50**		
	**0.75**		
**$KLaNb_2O_7$**		Ion Exchange from $RbLaNb_2O_7$ + KNO_3	380°C, 72 h
**$K_{1-x}Rb_x$**	**0.25**	Synthesis using stoichiometric amounts of $KLaNb_2O_7$ and $RbLaNb_2O_7$	400°C, 72 h
	**0.50**		
	**0.75**		
**$RbLaNb_2O_7$**		Direct Synthesis from $RbCO_3$, Nb_2O_5, and La_2O_3	850°C, 24 h; 1050°C, 48 h
**$Rb_{1-x}Cs_x$**	**0.25**	Synthesis using stoichiometric amounts of $RbLaNb_2O_7$ and $CsLaNb_2O_7$	400°C, 72 h
	**0.50**		
	**0.75**		
**$CsLaNb_2O_7$**		Direct Synthesis from $CsCO_3$, Nb_2O_5, and La_2O_3	1050°C, 48 h
**$KLaNb_2O_7$**		Ion Exchange from $RbLaNb_2O_7$ + KNO_3	380°C, 72 h
**$K_{1-x}Cs_x$**	**0.25**	Synthesis using stoichiometric amounts of $KLaNb_2O_7$ and $CsLaNb_2O_7$	600°C, 72 h
	**0.50**		
	**0.75**		
**$CsLaNb_2O_7$**		Direct Synthesis from $CsCO_3$, Nb_2O_5, and La_2O_3	1050°C, 48 h

*All reactions carried out in air.

2. Parent $ALaNb_2O_7$ (A = Li, Na, K, Rb, Cs) X-ray Diffraction data

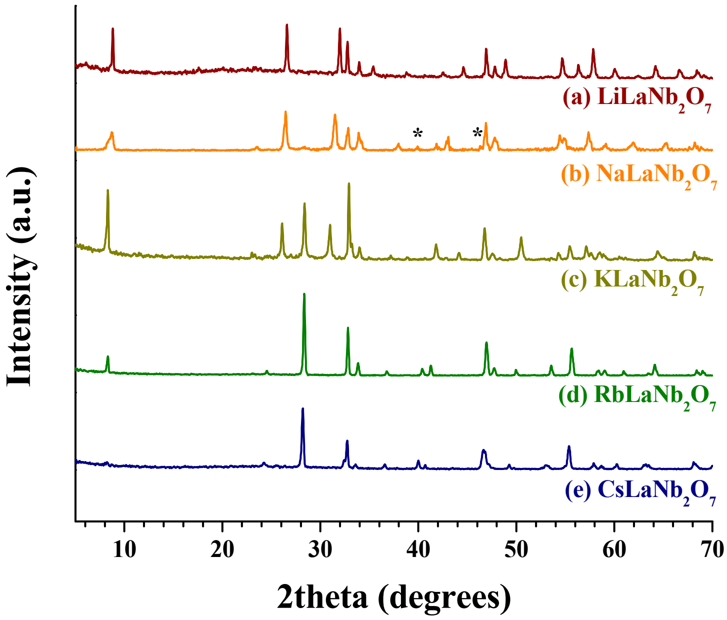

Figure S1. X-ray diffraction data for $ALaNb_2O_7$ for (a) $LiLaNb_2O_7$, (b) $NaLaNb_2O_7$ (taken at 300°C, *indicates Pt peaks from High-temperature XRD stage), (c) $KLaNb_2O_7$, (d) $RbLaNb_2O_7$, and (e) $CsLaNb_2O_7$.

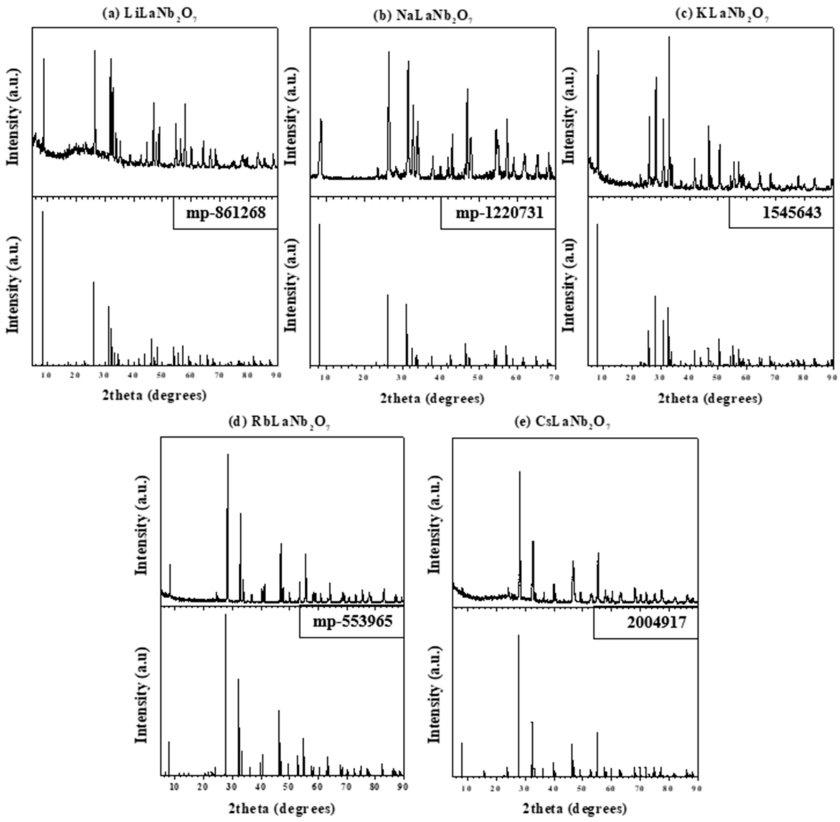

Figure S2. X-ray diffraction data (above) with reference patterns (below) for parent compounds, $ALaNb_2O_7$ (A = Li, Na*, K, Rb, Cs) (*collected at 300°C).

Table S2. Unit cell parameters for $ALaNb_2O_7$ (A = Li, Na, K, Rb, Cs) parent compounds.

Unit Cell Parameters for $ALaNb_2O_7$ Parent Compounds										
A	Experimental Values					Literature Values				
	a (Å)	b (Å)	c (Å)	V (Å^3)	Layer**	a (Å)	b (Å)	c (Å)	V (Å^3)	Ref.
**Li**	3.893(2)	-	20.435(8)	309.8(2)	S	3.8799(1)	-	20.3606(5)	306.50	[8]
**Na***	3.900(4)	-	21.183(2)	322.2(6)	S	3.9022(1)	-	21.1826(8)	322.55	[9]
**K**	3.899(1)	21.658(8)	3.888(1)	328.4(2)	PS	3.9060(1)	21.6030(7)	3.8879(1)	328.07	[10]
**Rb**	3.8858(3)	-	10.991(1)	165.95(3)	E	3.885(2)	-	10.989(3)	165.86	[6]
**Cs**	3.906(1)	-	11.160(3)	170.26(8)	E	3.908(1)	-	11.160(4)	170.44	[7]

*To eliminate any possible hydration, XRD data were collected at 300 °C. **Layer orientation: staggered (S), partially staggered (PS), and eclipsed (E).

3. Solid Solution $A_{1-x}A'_xLaNb_2O_7$ (A/A' = Li, Na, K, Rb, Cs) X-ray Diffraction Data

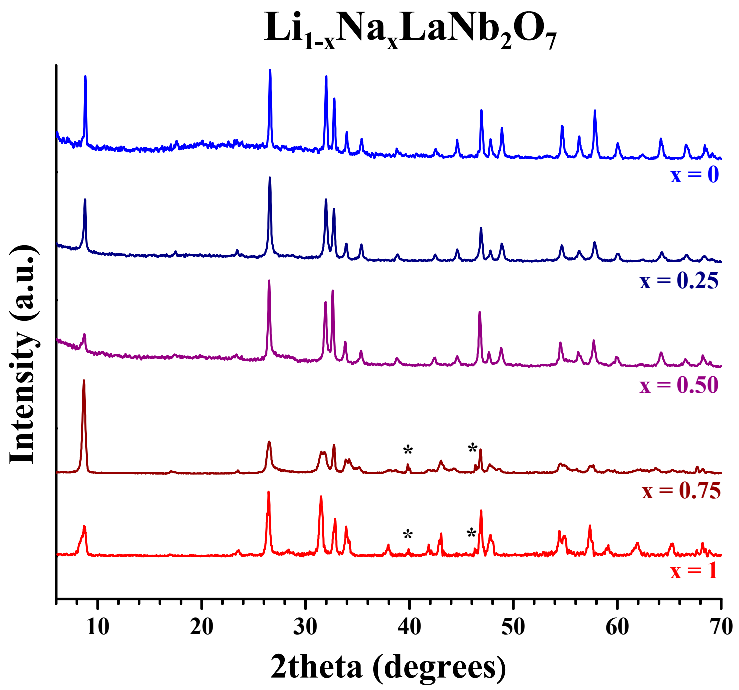

Figure S3. X-ray diffraction data for $Li_{1-x}Na_xLaNb_2O_7$ ($0 \le x \le 1$) solid solution series. Data for x = 0.75 and x = 1 were collected at 300°C. (* indicate Pt peaks from HTXRD stage).

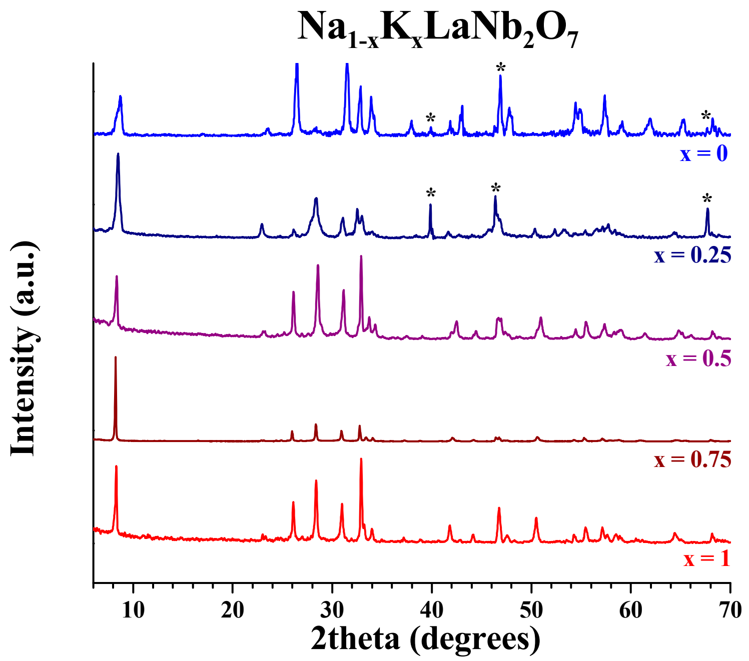

Figure S4. X-ray diffraction data for $Na_{1-x}K_xLaNb_2O_7$ ($0 \leq x \leq 1$) solid solution series. Data for x = 0 and x = 0.25 were collected at 300°C (* indicate Pt peaks from HTXRD stage).

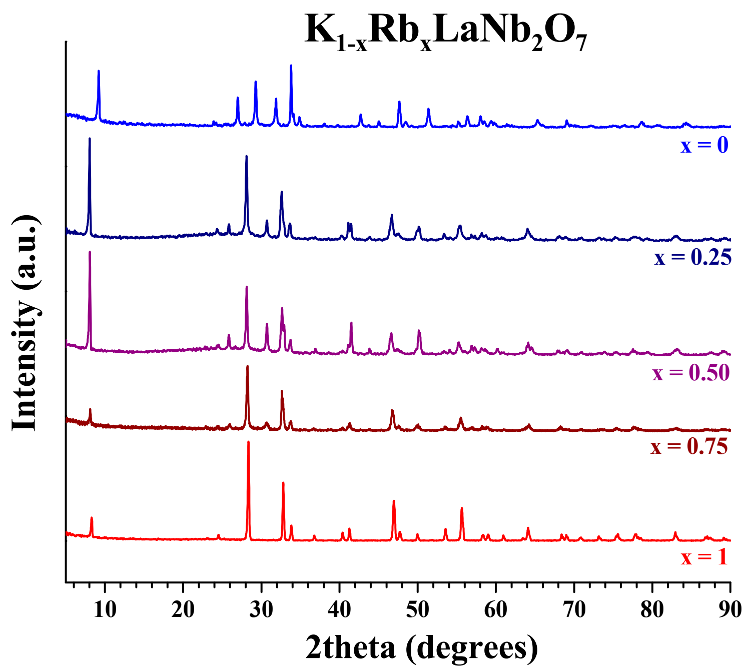

Figure S5. X-ray diffraction data for $K_{1-x}Rb_xLaNb_2O_7$ ($0 \leq x \leq 1$) solid solution series.

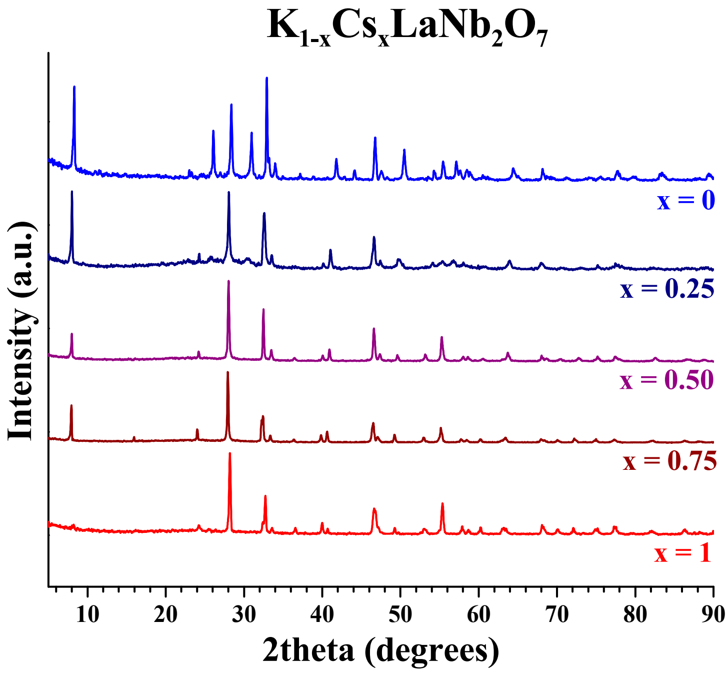

Figure S6. X-ray diffraction data for $K_{1-x}Cs_xLaNb_2O_7$ ($0 \le x \le 1$) solid solution series.

4. DSC Data of Parent Compounds, $ALaNb_2O_7$ (A = Li, Na, K) and $Li_{1-x}Na_xLaNb_2O_7$ ($0 \leq x \leq 1$)

Figure S7 shows DSC data of $ALaNb_2O_7$ (A = Li, Na, K). The metastable compounds $LiLaNb_2O_7$ and $NaLaNb_2O_7$ showed characteristic exothermic decomposition peaks at 719 °C and 812 °C respectively, while $KLaNb_2O_7$ showed no decomposition over the range of temperatures tested.

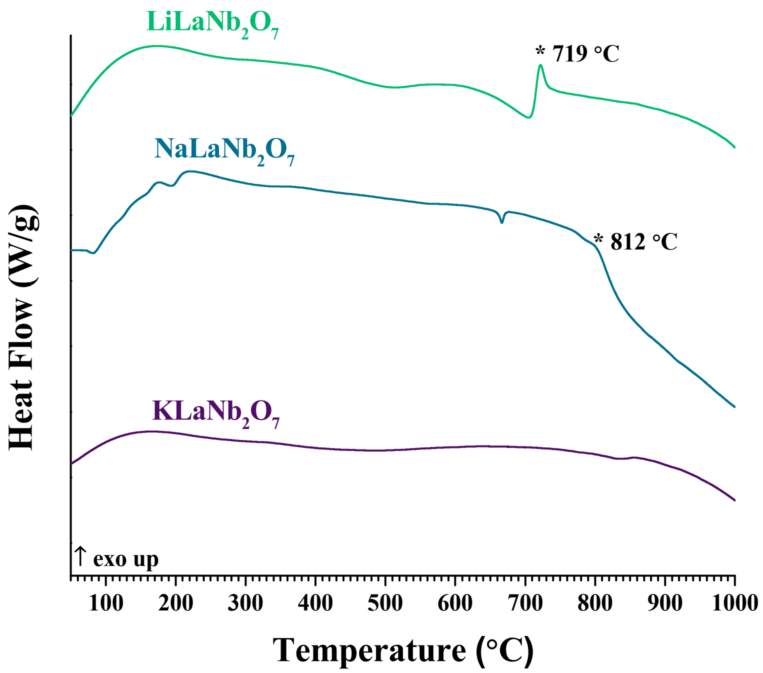

Figure S7. DSC data for parent compounds $ALaNb_2O_7$ (A = Li, Na, K) from room temperature to 1000 °C at a heating rate of 5°C/min under Ar atmosphere. Decomposition temperatures (*) are shown for $LiLaNb_2O_7$ and $NaLaNb_2O_7$. Small low temperature endotherms (< 200 °C) for $NaLaNb_2O_7$ are due to the loss of water of hydration and the endotherm at 680 °C has been attributed to a structure change in the perovskite layer (Sato et al., *Solid State Ionics* 1992, 57, 285-293). $KLaNb_2O_7$ is stable above 1000 °C. ($KLaNb_2O_7$, as well as $RbLaNb_2O_7$ and $CsLaNb_2O_7$, are all synthesized above 1000 °C.)

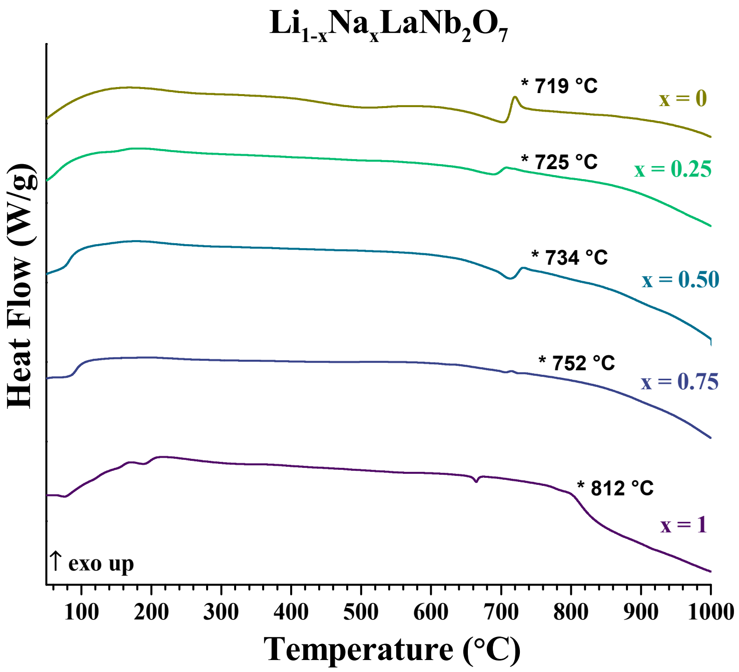

Figure S8. DSC data of $Li_{1-x}Na_xLaNb_2O_7$ solid solution series from room temperature to 1000 °C at a heating rate of 5°C/min under Ar atmosphere.

5. X-ray Powder Diffraction of Thermal Decomposition Products

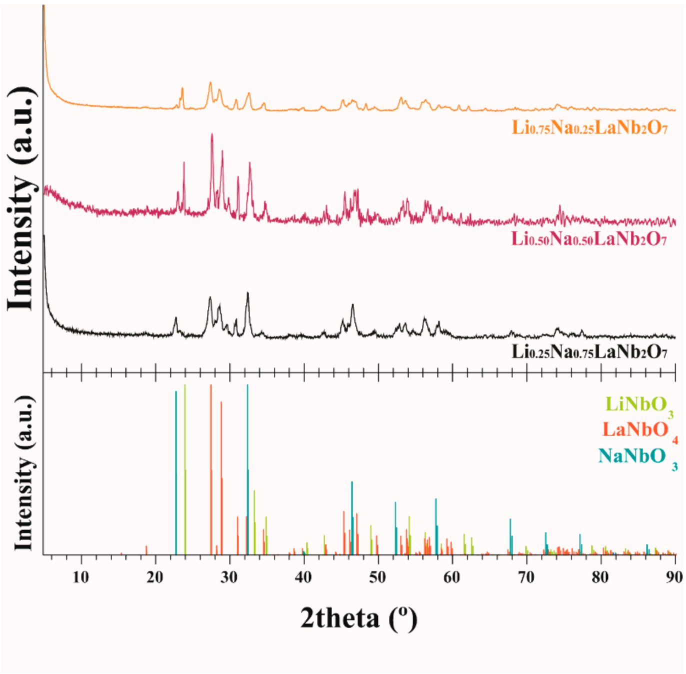

Figure S9. X-ray powder diffraction data for decomposition products for the series $Li_{1-x}Na_xLaNb_2O_7$. These are shown versus references for $LiNbO_3$, $NaNbO_3$ and $LaNbO_4$.

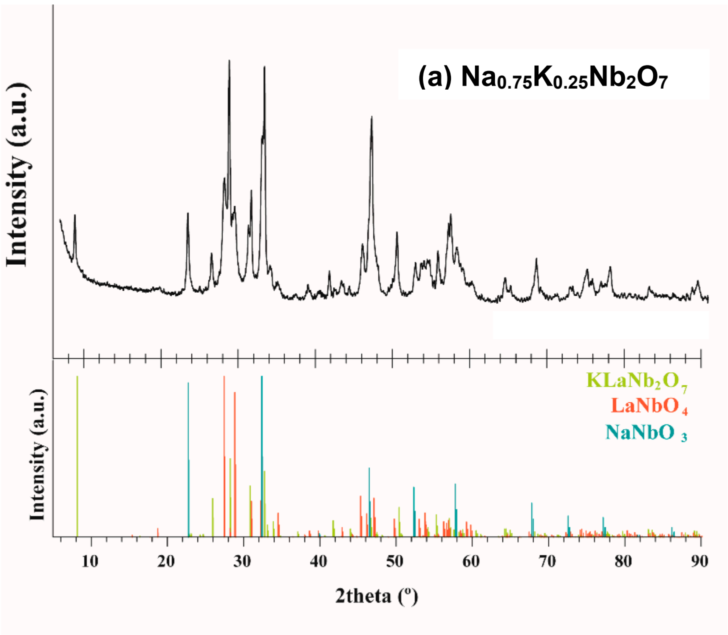

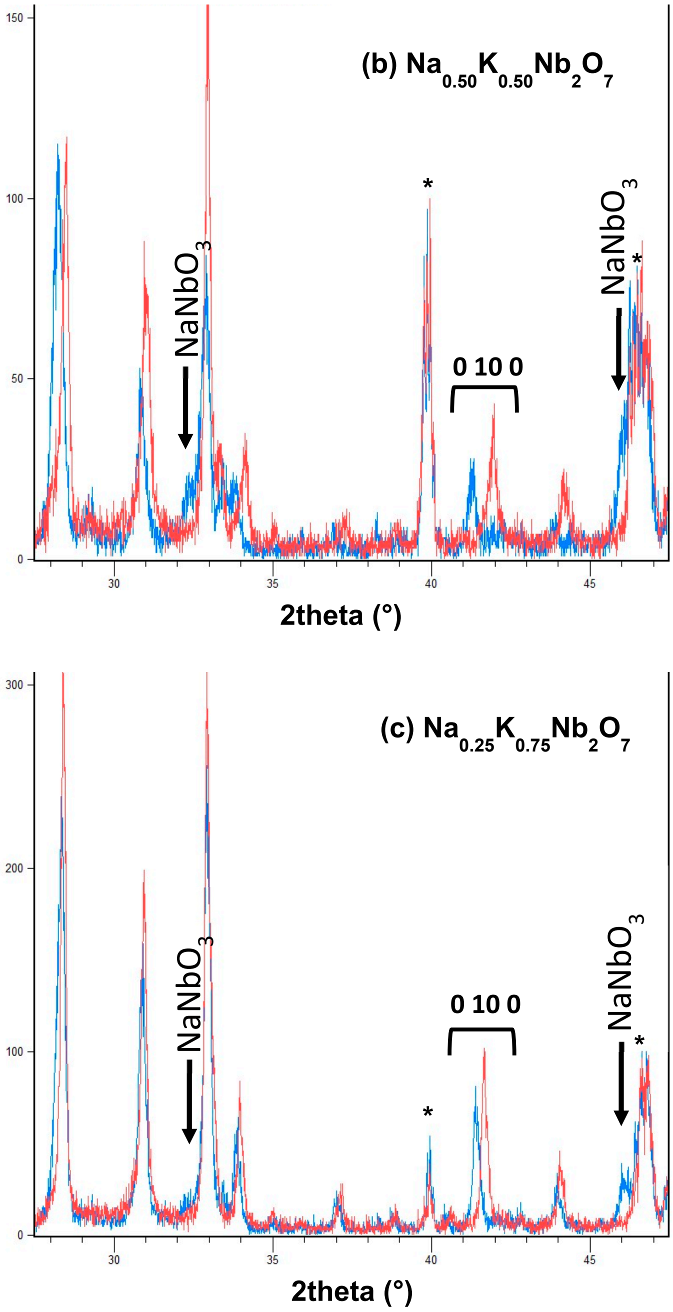

Figure S10. (a) X-ray powder diffraction data for $Na_{0.75}K_{0.25}LaNb_2O_7$ decomposition products relative to $NaNbO_3$, $LaNbO_4$, and $KLaNb_2O_7$. **(b,c)** Diffraction data from HTXRD at 500 °C, before heating (red) and after heating to 1000 °C (blue); **(b)** $Na_{0.50}K_{0.50}LaNb_2O_7$ and **(c)** $Na_{0.25}K_{0.75}LaNb_2O_7$; crystalline decomposition products are much less apparent than in (a), however, the shift of the 0 10 0 reflection pertinent to the perovskite layer spacing can be seen. (* indicates Pt peak from high temperature stage.)

6. Raman Spectroscopy Data of Parent Compounds $ALaNb_2O_7$ (A = Li, Na, K, Rb, Cs) and $A_{1-x}A'_xLaNb_2O_7$ (A/A' = Li, Na, K, Rb, Cs)

Table S3. Summary Raman data of apical Nb-O bond within the $A_{1-x}A'_xLaNb_2O_7$ (A/A′ = Li, Na, K, Rb, Cs; 0 ≤ x ≤ 1) solid solutions compounds.

Compound		Apical Nb-O bond vibration (cm^{-1})
$LiLaNb_2O_7$		943
$Li_{1-x}Na_x$	0.25	939
	0.50	934
	0.75	933
$NaLaNb_2O_7$		933
$Na_{1-x}K_x$	0.25	927
	0.50	926
	0.75	925
$KLaNb_2O_7$		929
$K_{1-x}Rb_x$	0.25	927
	0.50	928
	0.75	927
$RbLaNb_2O_7$		930
$Rb_{1-x}Cs_x$	0.25	928
	0.50	927
	0.75	927
$CsLaNb_2O_7$		926
$KLaNb_2O_7$		929
$K_{1-x}Cs_x$	0.25	927
	0.50	927
	0.75	927
$CsLaNb_2O_7$		926

Raman spectra for all parent compounds are shown in **Figure S11,** while the solid solutions are shown in **Figures S12 - S16.**

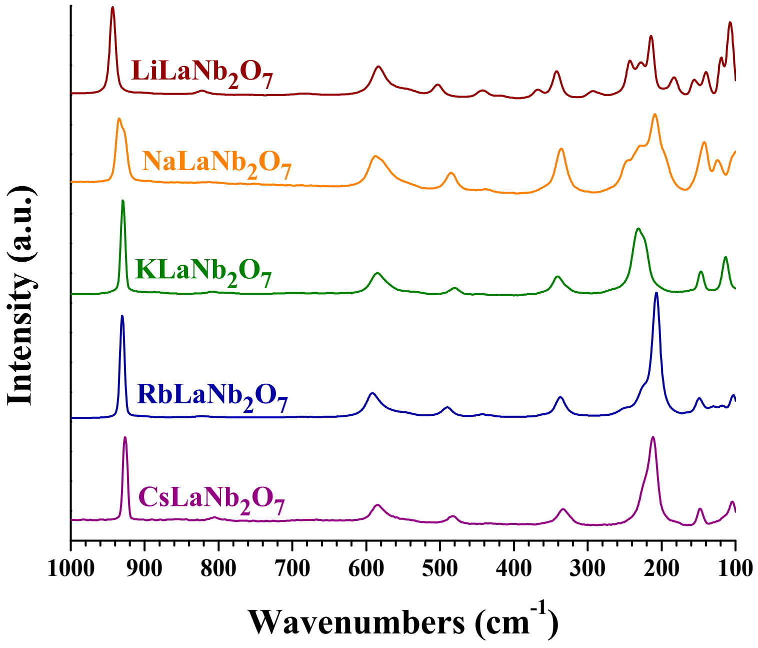

Figure S11. Raman spectra for parent compounds $ALaNb_2O_7$ (A = Li, Na, K, Rb, Cs).

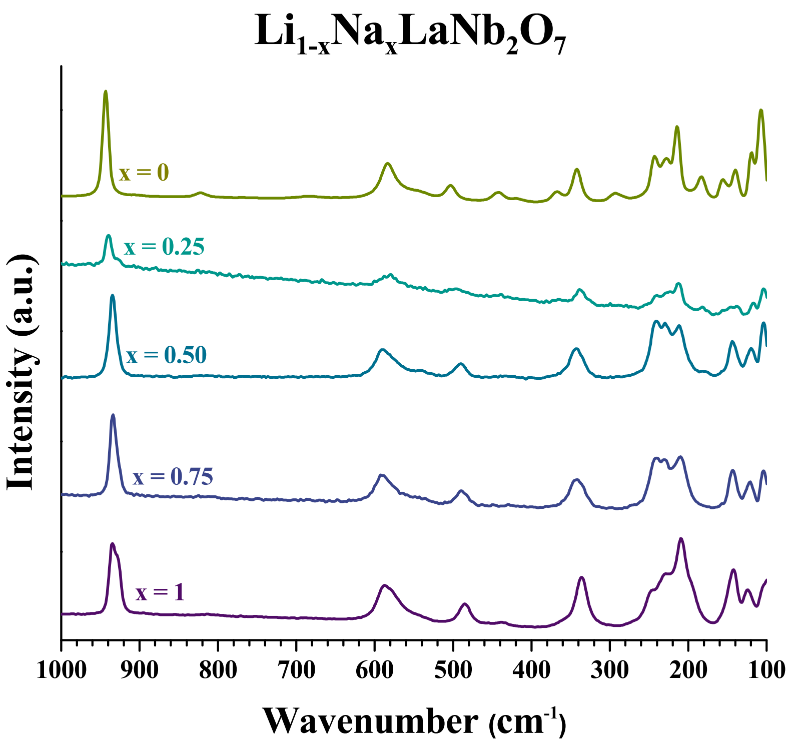

Figure S12. Raman spectra for $Li_{1-x}Na_xLaNb_2O_7$ ($0 \leq x \leq 1$) solid solution series.

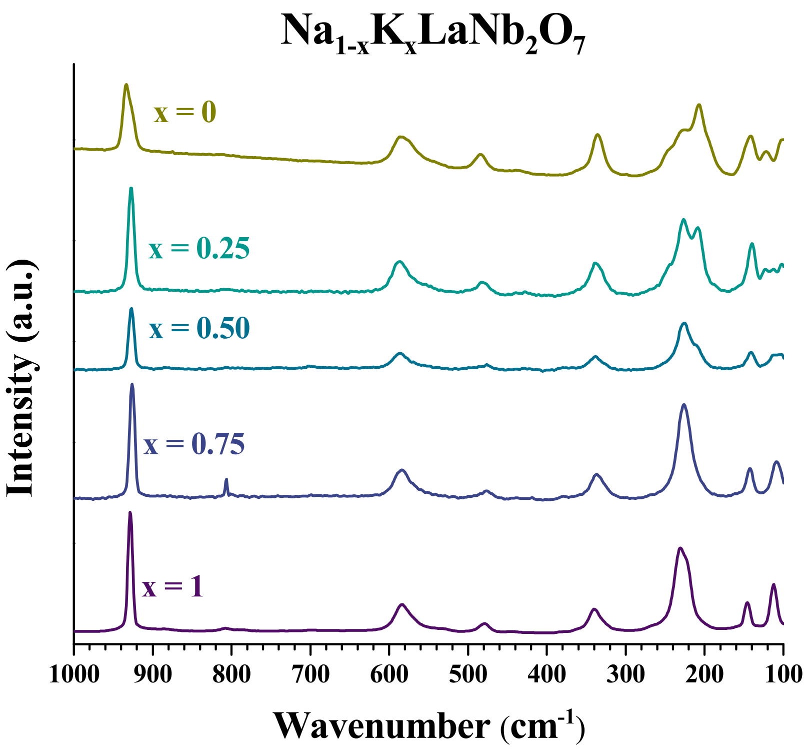

Figure S13. Raman spectra for $Na_{1-x}K_xLaNb_2O_7$ ($0 \leq x \leq 1$) solid solution series.

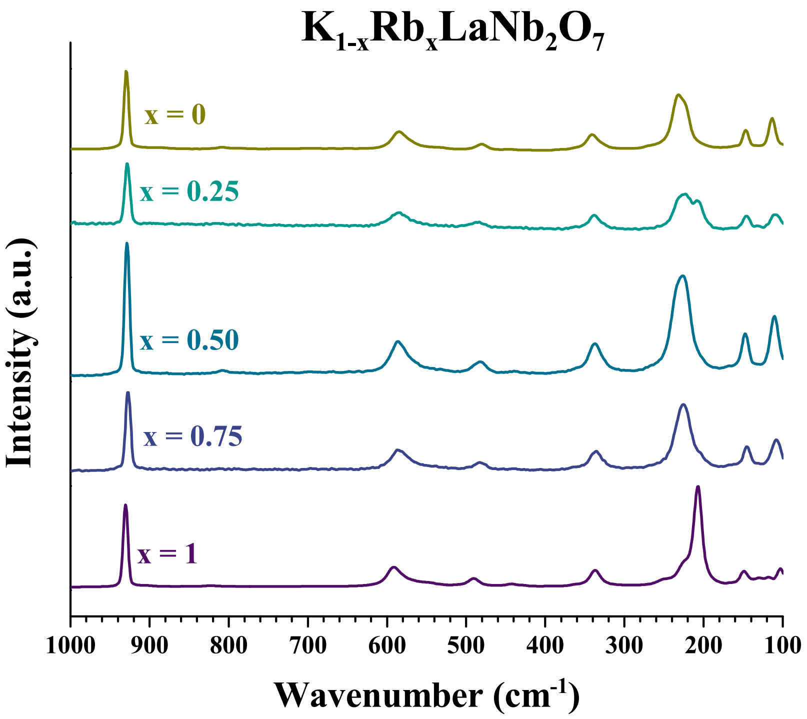

Figure S14. Raman spectra for $K_{1-x}Rb_xLaNb_2O_7$ ($0 \leq x \leq 1$) solid solution series.

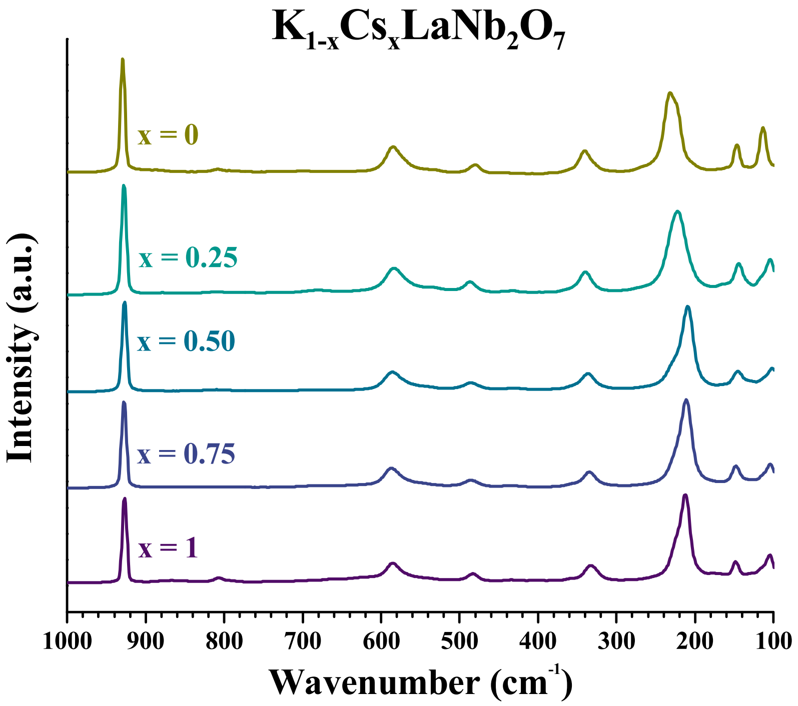

Figure S15. Raman spectra for $K_{1-x}Cs_xLaNb_2O_7$ ($0 \leq x \leq 1$) solid solution series.

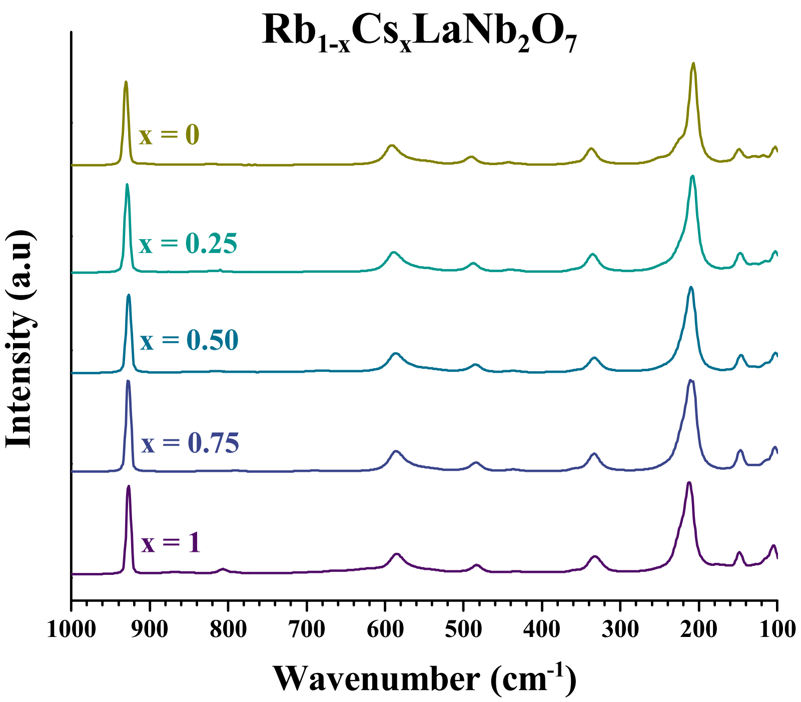

Figure S16. Raman spectra of $Rb_{1-x}Cs_xLaNb_2O_7$ ($0 \leq x \leq 1$) solid solution series.
